# Improved clinical outcome using transarterial chemoembolization combined with radiofrequency ablation for patients in Barcelona clinic liver cancer stage A or B hepatocellular carcinoma regardless of tumor size: results of a single-center retrospective case control study

**DOI:** 10.1186/s12885-019-6237-5

**Published:** 2019-10-22

**Authors:** Yanqiao Ren, Yanyan Cao, Hong Ma, Xuefeng Kan, Chen Zhou, Jiacheng Liu, Qin Shi, Gansheng Feng, Bin Xiong, Chuansheng Zheng

**Affiliations:** 1 Department of Radiology, Union Hospital, Tongji Medical College, Huazhong University of Science and Technology, Wuhan, 430022 China; 2 Hubei Key Laboratory of Molecular Imaging, Wuhan, 430022 China; 3 Cancer Center, Union Hospital, Tongji Medical College, Huazhong University of Science and Technology, 1277 JieFang Avenue, Wuhan, 430022 Hubei China

**Keywords:** Chemoembolization, Therapeutic, Radiofrequency ablation, Hepatocellular carcinoma

## Abstract

**Background:**

To determine the safety and efficacy of transarterial chemoembolization (TACE) combined with radiofrequency ablation (hereafter, TACE-RFA) in treating Barcelona Clinic Liver Cancer (BCLC) Stage A or B (hereafter, BCLC A/B) hepatocellular carcinoma (HCC) patients, and to explore the range of tumor sizes suitable for combination therapy.

**Methods:**

This retrospective study assessed the consecutive medical records of HCC patients with BCLC A/B who received TACE-RFA or TACE from September 2009 to September 2018. Progression-free survival (PFS), overall survival (OS), therapeutic response, and complications were compared between the two groups.

**Results:**

Among 2447 patients who received TACE-RFA or TACE, 399 eligible patients were enrolled in our study, including 128 patients in the TACE-RFA group and 271 patients in the TACE group. Compared with the TACE group, the PFS and OS rates of 1,3,5,8 years in the TACE-RFA group were significantly better, with higher objective tumor regression rate and better disease control rate. RFA treatment did not increase the risk of death in patients with HCC, and both liver subcapsular hematoma and bile duct injury were improved by symptomatic treatment. Serum α-fetoprotein level and treatment method were important independent prognostic factors for OS, whereas albumin, hepatitis B and treatment method were important independent prognostic factors for PFS. Subgroup analysis showed that patients in the TACE-RFA group always showed better OS and PFS.

**Conclusions:**

TACE-RFA had an advantage over TACE alone in prolonging PFS and improving OS in HCC patients with BCLC A/B, and can benefit patients regardless of tumor size.

## Background

Hepatocellular carcinoma (HCC) is still one of the most common tumors in the world and the second most common cause of cancer death [1, 2]. Although ultrasound and serum alpha-fetoprotein (AFP) levels monitor high-risk populations for early detection of HCC, most patients are diagnosed as advanced stages [3–5]. Moreover, some cirrhosis patients with insufficient liver reserves may not be suitable for hepatectomy [6, 7]. Liver transplantation is expensive and donors are often scarce, and patients need to meet strict screening criteria. Only a few patients can receive liver transplantation [8]. As a result, only 30% or less of HCC patients are able to benefit from curative therapies [9, 10].

In recent years, transarterial chemoembolization (TACE) as a palliative therapy has been recognized as the standard method for patients with unresectable HCC [11, 12]. TACE can improve survival by combining targeted chemotherapy with ischemic necrosis caused by arterial embolization [12, 13]. A meta-analysis indicated that TACE procedure had significant survival benefits for HCC patients who cannot undergo surgery [10]. However, many research reports that TACE alone is difficult to cause complete tumor necrosis even if the tumor diameter is small [11, 14].

Radiofrequency ablation (RFA), which kills tumors through thermal damage, is considered an effective local treatment for HCC [12, 15, 16]. Some studies suggested that RFA alone had excellent survival results for small HCCs [17–19]. However, tumor recurrence after ablation is still a major challenge in the treatment of small HCCs by RFA [20]. Furthermore, RFA has a limited range, and for large HCCs, complete ablation is difficult to achieve [6, 11, 21].

Given the limitations of these two local treatments, TACE-RFA combination therapy may be able to control tumor progression and prolong survival of HCC patients. And according to Barcelona Clinic Liver Cancer (BCLC) guideline, RFA is mainly applicable to stage A HCC patients, while TACE is recommended as the first-line treatment for stage B HCC patients [22, 23]. Hence, the purpose of this retrospective study was to evaluate the safety and efficacy of combination therapy of TACE and RFA for patients with stage A/B HCC. Moreover, RFA is generally considered to be suitable for tumors no more than 5 cm in size [24]. Thus, this study also explored the tumor diameters suitable for the combined treatment.

## Methods

### Study design and patient selection

This retrospective study received local hospital ethic committee approval. Written informed consent was obtained from all patients prior to treatment.

From September 2009 to September 2018, 2447 consecutive patients with HCC underwent combination therapy using TACE and RFA (TACE followed by RFA) or TACE alone in our medical center. Prior to these patients underwent initial TACE, the treatment strategy was recommended by the multidisciplinary tumor board. If patients chose combination therapy, the time of RFA after TACE was dependent on the disappearance of syndrome after embolization and the recovery of liver function, and in our center, RFA was usually performed 1 to 2 weeks after TACE. Patients who rejected RFA were only treated with TACE.

**Fig. 1:**
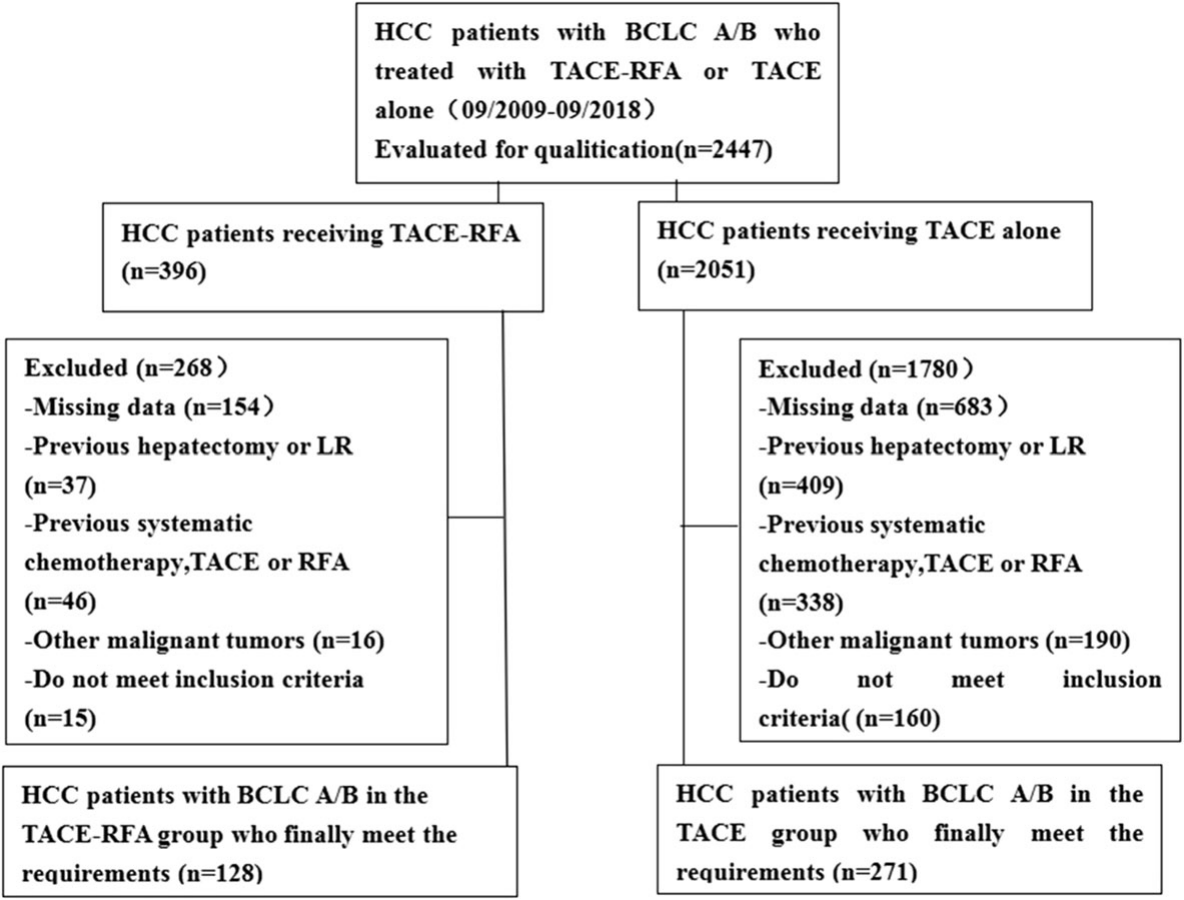
Flow chart shows the screening procedure for patients with Barcelona Clinic Liver Cancer (BCLC) A/B hepatocellular carcinoma (HCC)

The diagnosis of HCC depended on the diagnostic criteria of the European Association for the Study of Liver (EASL) and the American Association for the Study of Liver Disease [25]. A total of 399 patients who met the eligibility criteria were included in this study:(1) Child-Pugh class A or B; (2) the number of tumors <= 3; (3) liver resection or transplantation was denied; (4) no evidence of invasion into the portal or hepatic venous branches, extrahepatic metastasis, or uncontrolled ascites; (5) BCLC A/B. The patient was excluded if the exclusion criteria were met: (1) had previously undergone any treatment for HCC; (2) had renal failure, cardiac failure or hemorrhagic risk; (3) had other malignancies besides HCC (Fig. 1).

### TACE

Transarterial chemoembolization was performed according to our institutional standard protocol and has been previously reported [26, 27]. In short, tumor staining, and tumor feeding arteries were determined by angiographies, then, a 2.6-Fr microcatheter (Terumo, Japan) was inserted into the tumor donor arteries as superselectively as possible. First, an emulsion of 2–20 mL iodized oil (Lipiodol Ultra-Fluid; Laboratoire Andre Guerbet, Aulnay-sous-Bois, France) and 20–60 mg adriamycin was administered into the target vessels. Then it was embossed with gelatin sponge particles (300–700 um, Cook, Bloomington, Indiana, USA).

### RFA

The RFA procedure was performed in accordance with the standard treatment regimen stated in our previous study [26]. In brief, after analgesia (10 mg of morphine) and local anesthesia (5–10 ml of lidocaine), the electrode needle was inserted into the tumor nodule under the guidance of ultrasound or computed tomography (CT). RITA 1500 generator (RITA Medical Systems Inc., Mountain View, USA) was used. For tumors <= 2.0 cm in diameter, a single extendable electrode was placed into the tumor center, otherwise multi-hook probe was used. And to attain a safe range of 0.5–1.0 cm, multiple overlapping ablation zones were required.

### Definition and evaluation of data

Progression-free survival (PFS) and overall survival (OS) were compared between RFA and TACE groups. We defined the time from first TACE procedure to date of disease progression as PFS. OS referred to the interval between the first TACE procedure and either death or last follow-up. Modified Response Evaluation Criteria in Solid Tumors (mRECIST) was used to evaluate treatment response 1 month after treatment. Complete response (CR) refers to the absence of enhancement in all target lesions; partial response (PR) is classified as at least a 30% decrease in the sum of the diameters of viable tumors; progressive disease (PD) is an increase of at least 20% in the sum of the diameters of target lesions; stable disease (SD) refers to any cases that do not qualify for either PR or PD [28]. Objective tumor regression referred to CR or PR. Disease control rate represented CR, PR or SD. Using the Society of Interventional Radiology classification system to evaluate the safety of TACE or RFA in both groups [29]. Major complications were defined as events leading to death and disability.

### Follow-up

Laboratory tests, contrast-enhanced CT or magnetic resonance (MR) imaging examination were performed 1 month after initial TACE. Imaging (contrast-enhanced CT or MR) and laboratory examinations were performed every 2–3 months for patients, follow-up continued until the patient died or the end point of this study’s follow-up.

### Statistical analyses

All analyses were performed using R language version 3.3.3, and *P* < 0.05 was considered statistically significant. Quantitative data were represented by^−^x ± s and discrete variables were represented by proportion. Quantitative data were performed by Student’s t-test, and Chi-squared test was used to categorical data. Kaplan-Meier method and log-rank test were performed to evaluate the differences in PFS and OS between the two groups. Cox proportional hazards regression model was used to analyze possible prognostic factors affecting PFS and OS. For the selection of multivariate analysis variables, according to the Akaike information criterion, the final stepwise regression variables were screened out using the lowest score. And sex and age were always retained in the model to correct for confounding factors. Conditional tree model was applied to explore the tumor size suitable for combined treatment.

## Results

### Study population and patient characteristics

From September 2009 to September 2018, a total of 2447 patients received TACE-RFA or TACE alone, and 2048 patients were excluded because they did not meet the study requirements, as shown in Fig. 1. Finally, a total of 399 patients were included in this study, 128 were treated with combination therapy and 271 were treated with TACE alone. The detailed clinical characteristics of the 399 patients are summarized in Table 1.

**Table 1:** Baseline characteristics of patients in the TACE group and the TACE-RFA group

Characteristic	TACE group (*n* = 271)	TACE-RFA group (*n* = 128)	*P* value
Sex			0.242
Male	216 (79.7%)	109 (85.2%)	
Female	55 (20.3%)	19 (14.8%)	
Age (years)			0.476
Mean value	56.1 ± 10.8	55.3 ± 10.4	
Range	28–79	16–83	
Albumin g/dL	37.8 ± 4.9	38.0 ± 5.9	0.692
Total bilirubin u mol/L	20.1 ± 17.5	24.0 ± 54.4 0	0.435
Alpha-fetoprotein level			0.443
< =400 ng/mL	180 (66.4%)	80 (62.5%)	
> 400 ng/mL	91 (33.6%)	48 (37.5%)	
Child-Pugh score			0.676
A	239 (88.2%)	111 (86.7%)	
B	32 (11.8%)	17 (13.3%)	
BCLC stage			0.000
A	110 (40.6%)	88 (68.8%)	
B	161 (59.4%)	40 (31.2%)	
Liver disease type			0.504
Hepatitis B	242 (89.3%)	109 (85.2%)	
Hepatitis C	9 (3.3%)	6 (4.7%)	
Other	20 (7.4%)	13 (10.2%)	
Mean tumor diameter (cm)	6.8 ± 4.3	4.5 ± 3.1	0.000
Tumor diameter range (cm)			0.000
< =3.0	70 (25.8%)	50 (39.1%)	
3.1–5.0	50 (18.5%)	40 (31.2%)	
5.1–10.0	89 (32.8%)	27 (21.1%)	
> 10	62 (22.9%)	11 (8.6%)	
Number of tumors			0.102
1	201 (74.2%)	102 (79.7%)	
2	49 (18.1%)	23 (18.0%)	
3	21 (7.7%)	3 (2.3%)	

*BCLC* Barcelona Clinic Liver Cancer, *RFA* radiofrequency ablation, *TACE* transarterial chemoembolization

The median follow-up period was 38.1 months (range, 5.7–110.5 months) in the TACE-RFA group and 27.8 months (range,14.4–103.9 months) in the TACE group. In the TACE group, 171 patients died during the observation period, whereas in the TACE-RFA group, only 42 patients died.

### Treatment response

The objective tumor regression rate of patients in the TACE-RFA group was 85.9%, and that in the TACE group was 44.7%, which was statistically significant between the two groups. In addition, the disease control rate in the TACE-RFA group was 91.4%, and that in the TACE group alone was 72.0%. Hence, compared with the TACE group, the TACE-RFA group had better tumor response.

### Complications

In the TACE group, three patients had severe complications, with an incidence of 1.1%. One patient died 3 days after TACE due to acute liver and kidney failure, and two patients developed biloma after TACE. Two cases of severe complications in TACE-RFA group, with an incidence of 1.6%. Subcapsular hematoma of the liver was found on CT scan in 1 patient after multi-hook probe puncture, one patient had bile duct injury during RFA. There was no significant difference in the incidence of major complications between the two groups (*P* = 0.66).

### Overall survival

**Fig. 2:**
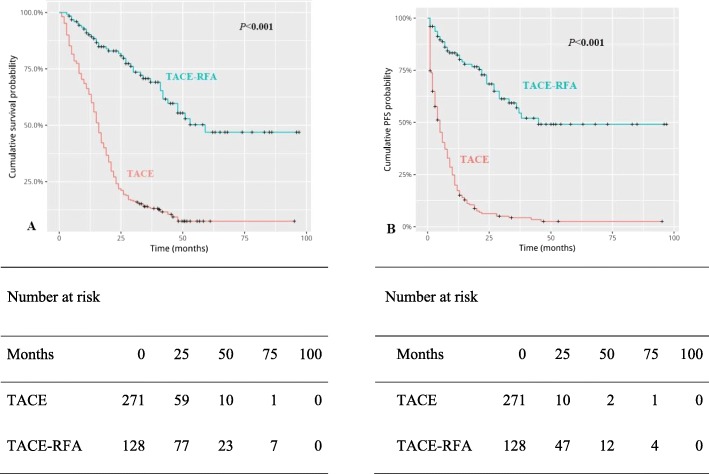
Kaplan-Meier curves of cumulative survival (**a**) and progression-free survival (PFS) (**b**) in hepatocellular carcinoma patients with Barcelona Clinic Liver Cancer (BCLC) A/B who received transarterial chemoembolization (TACE) and radiofrequency ablation (RFA) or TACE alone

Median OS was 59 months in the TACE-RFA group and 16 months in the TACE group(*P* < 0.001) (Fig. 2a). In the TACE-RFA group, the 1-, 3-, 5- and 8-year survival rates were 90.6, 76.6, 68.0, 68.0%. In the TACE group, the 1-, 3-, 5- and 8-year survival rates were 64.5, 15.1, 10.8, 10.8%. Univariable analyses showed that mean tumor size, total bilirubin, AFP > 400 ng/mL, BCLC B and therapy method (TACE-RFA) were related to OS (Table 2). Then, through multivariable analysis (Table 3), we found that AFP > 400 ng/mL was an independent risk factor for OS and TACE-RFA combination therapy was significantly in connection with better OS.

**Table 2:** Univariate analysis of prognostic factors for overall survival and progression-free survival

Factor	OS			PFS	
	HR	*P* Value		HR	*P* Value
Sex (female vs. male)	1.091	0.576		1.051	0.78
Age	1.008	0.162		1.004	0.489
Mean tumor diameter (cm)	1.102	< 0.001		1.119	< 0.001
Albumin g/dL	0.987	0.265		0.995	0.667
Total bilirubin u mol/L	1.004	0.002		1.002	0.139
AFP > 400 ng/mL	1.868	< 0.001		1.941	< 0.001
Child-Pugh B	0.847	0.445		0.833	0.437
BCLC B	2.072	< 0.001		2.631	< 0.001
Hepatitis B	1.589	0.118		1.203	0.534
TACE-RFA	0.205	0		0.147	0
Number of tumors	1.089	0.446		1.089	0.488

*OS* overall survival, *PFS* progression-free survival, *HR* hazard ratio, *AFP* alpha-fetoprotein, *BCLC* Barcelona Clinic Liver Cancer, *RFA* radiofrequency ablation, *TACE* transarterial chemoembolization

**Table 3:** Multivariate analysis of prognostic factors for overall survival and progression-free survival

Factor	OS			PFS	
	HR	*P* Value		HR	*P* Value
Sex (female vs. male)	0.976	0.925		0.668	0.156
Age	1.01	0.229		1.011	0.189
Mean tumor diameter (cm)	1.02	0.572		1.003	0.932
Albumin g/dL	1.002	0.915		1.053	0.027
Total bilirubin u mol/L	1.008	0.45		1.012	0.239
AFP > 400 ng/mL	1.755	0.006		1.456	0.082
BCLC B	1.563	0.109		0.28	0.104
Hepatitis B	0.898	0.737		0.433	0.012
TACE-RFA	0.461	0		0.213	< 0.001

*OS* overall survival, *PFS* progression-free survival, *HR* hazard ratio, *AFP* alpha-fetoprotein, *BCLC* Barcelona Clinic Liver Cancer, *RFA* radiofrequency ablation, *TACE* transarterial chemoembolization

### Progression-free survival

Median PFS was 45 months in the TACE-RFA group and 4 months in the TACE group (Fig. 2b). The cumulative PFS rates of 1, 3, 5, and 8 years in the TACE-RFA group were significantly higher than that in the TACE group. Univariable analyses indicated that mean tumor size, AFP > 400 ng/mL, BCLC B and TACE-RFA was related to PFS (Table 2). Multivariable analysis revealed that albumin, hepatitis B and TACE-RFA were associated with PFS (Table 3).

### Subgroup analysis by tumor size

**Fig. 3:**
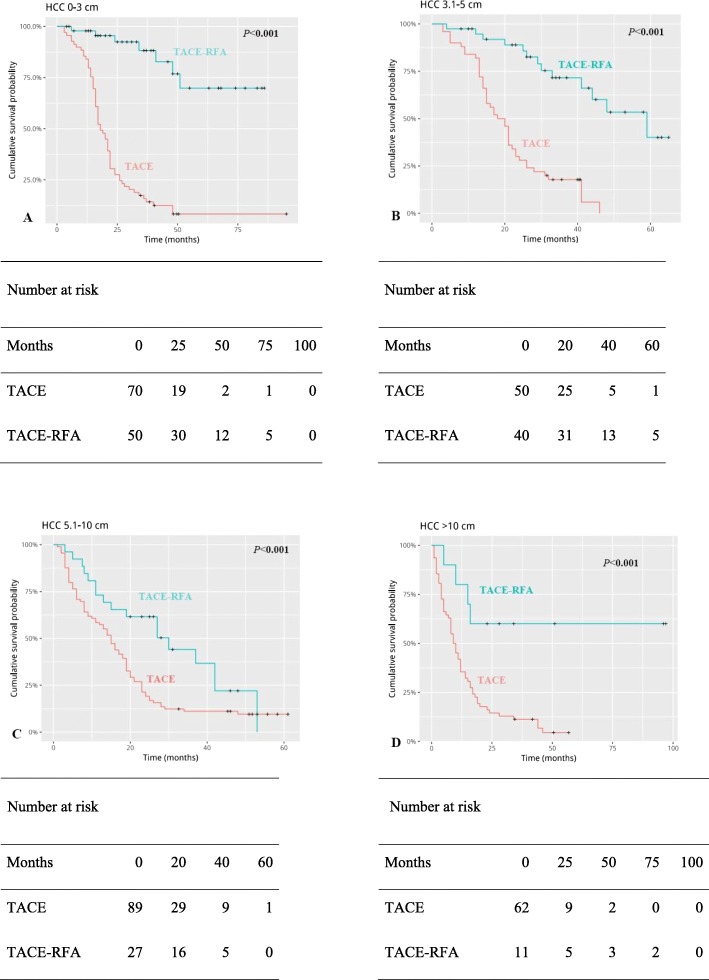
Kaplan-Meier curves of cumulative survival of four groups (**a**-**d**) of Barcelona Clinic Liver Cancer (BCLC) A/B hepatocellular carcinoma patients grouped by tumor size

**Fig. 4:**
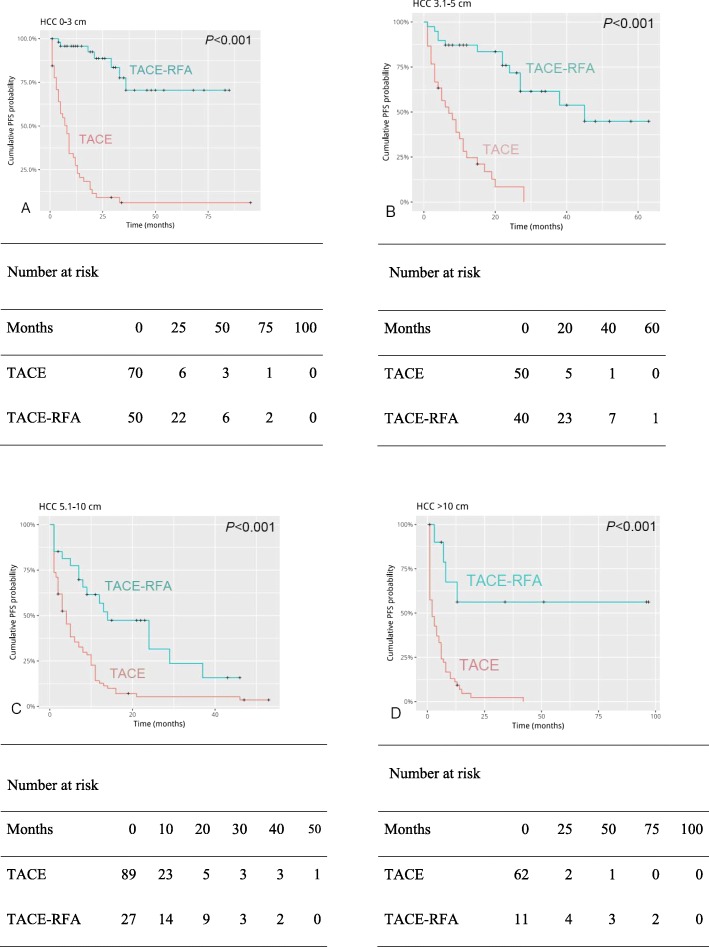
Kaplan-Meier curves of progression-free survival (PFS) of four groups (**a**-**d**) of Barcelona Clinic Liver Cancer (BCLC) A/B hepatocellular carcinoma patients grouped by tumor size

In the subgroup analysis, for HCC patients with tumor diameter less than 3 cm, the cumulative OS rates (Fig. 3a) and cumulative PFS rates (Fig. 4a) at 1, 3, 5,8 years were better in patients treated with TACE-RFA than those treated with TACE alone. For HCC patients with tumor diameter of 3–5 cm, there were no difference in 1-year cumulative OS rates between the two groups (Fig. 3b). The cumulative PFS rates of 1,3,5,8 years were different between the two groups (Fig. 4b).

Similarly, the 1,3,5,8 years cumulative OS rates (Fig. 3c) and PFS rates (Fig. 4c) of HCC patients in the TACE-RFA group with tumor diameter of 5-10 cm were significantly better than those in the TACE group. And it was the same with patients with tumor diameter greater than 10 cm (OS: Fig. 3d; PFS: Fig. 4d).

### Death risk curve

**Fig. 5:**
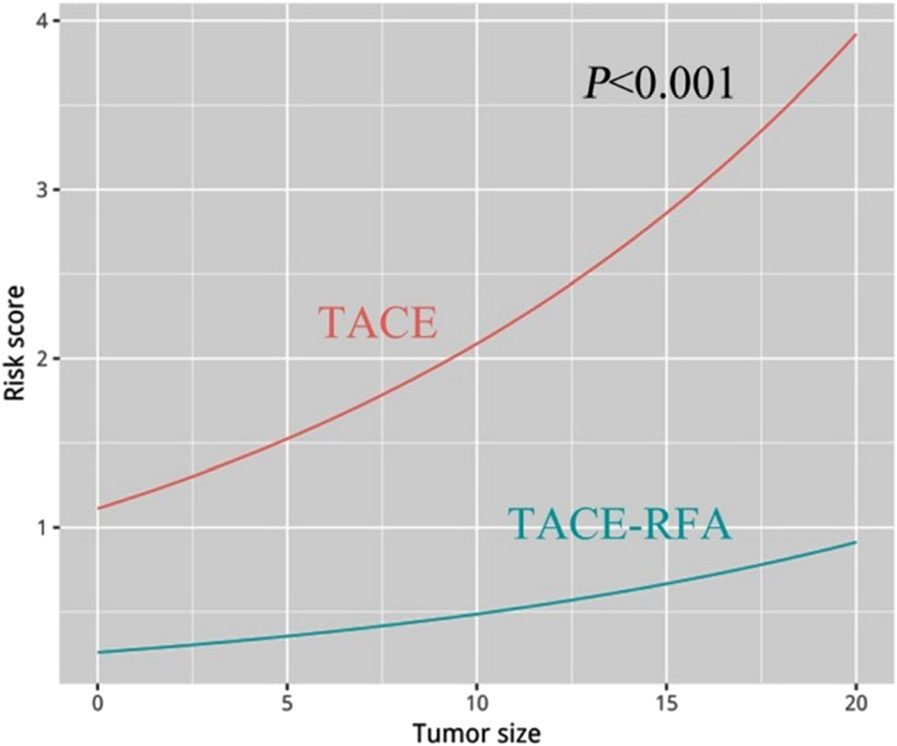
Death risk curve of Barcelona Clinic Liver Cancer (BCLC) A/B hepatocellular carcinoma patients treated with transarterial chemoembolization (TACE) and radiofrequency ablation (RFA) or TACE alone

As the tumor size increases, the death risk curve of the TACE group increased significantly faster than the TACE-RFA group, and the difference in risk score between them also increased (Fig. 5). In other words, the larger the tumor size, the greater the reduction in death risk in the TACE-RFA group.

## Discussion

The combination of TACE and RFA has the following theoretical advantages [11, 20, 30, 31]: (1) TACE procedure causes decreased hepatic artery blood flow, which reduces the heat sink effects and increases the ablation zone; (2) Satellite lesions can be detected through TACE, which is more beneficial to RFA. Therefore, the combination of TACE and RFA was supposed to improve survival of HCC patients.

The results of this study showed that TACE-RFA had better efficacy in the treatment of BCLC A/B HCC, which was mainly manifested as objective tumor regression rate, disease control rate, OS and PFS rates of patients with 1,3,5,8 years were significantly better than TACE alone. Similar to our results, Hyun et al. [32] concluded that for HCC patients with tumor size < 3 cm, the TACE-RFA group had a better cumulative survival rates of 1,2,3 years than the TACE group, while our results showed that the TACE-RFA group still showed good survival benefits for HCC patients with medium - or large-diameter tumors. Meanwhile, a study [20] has reported that TACE combined with RFA in the treatment of early HCC is more effective than RFA alone. This indicates that combined therapy, as described by the theoretical advantages, has produced certain synergistic therapeutic effect and can improve the therapeutic efficacy of patients with HCC.

It is reported that AFP level is related to tumor activity and play an important role in the diagnosis and prognosis of HCC patients [33]. In our study, AFP > 400 ng/mL was a bad prognostic factor for OS. Tang et al. [11] noted that treatment modality (TACE- RFA) is an independent prognostic factor affecting the survival of patients with unresectable HCC. Meanwhile, Hyun et al. [32] reported that TACE alone was the only risk factor affecting the survival of early HCC. The results of our study suggested that TACE-RFA was the only protective factor for OS. One study reported [34] that TACE, serum bilirubin, and tumor diameter were prognostic factors of PFS. Therefore, serum AFP and treatment modality may be important factors affecting prognosis.

TACE procedure results in intratumoral ischemia and hypoxia, and tumor progression may be caused by up-regulation of angiogenic factors [22]. Furthermore, large tumors are sometimes supplied by extrahepatic collateral pathways, leading to incomplete tumor necrosis [35]. Therefore, the larger the tumor diameter, the more difficult TACE alone is to achieve complete tumor necrosis. At this time, RFA may be able to make up for the deficiency caused by TACE alone by ablation of residual tumor areas with poor iodine-oil deposition. This may explain our results: the death risk curve of the TACE group was significantly higher than that of the TACE-RFA group.

Currently, the range of tumor diameter in HCC patients who are eligible for RFA is still controversial. Many studies [16, 17, 19] have shown that RFA is a curative local treatment for small HCCs, and due to limited ablation range and coagulative necrosis size, RFA is not recommended for HCCs with a diameter greater than 5 cm [23, 24]. However, the results of our subgroup analysis by tumor size showed that four groups of patients who received TACE-RFA showed better OS and PFS. Therefore, if RFA is available after physicians’ evaluation, we should actively recommend RFA treatment to patients, regardless of the tumor size.

In recent years, charged particle therapy (CPT), a relatively novel, non-invasive and promising treatment option, has made remarkable progress in the treatment of HCC [36–38]. Protons (proton beam therapy, PBT) and carbon ions (carbon ion therapy, CIT) are the most commonly used particles of CPT. Chiba et al. [39] noted that for HCC patients receiving PBT, there was no statistically significant difference in the local control rate at 5 years between patients with tumors < 5 cm in maximal diameter and those with > 5 cm, indicating that PBT was also applicable for patients with large HCCs. One study [40] showed that for HCC patients receiving CIT or TACE, patients in the CIT group were significantly better than those in the TACE group in terms of 3-year OS, PFS and local control rates. However, although many series and some trials have reported the efficacy of CPT, there is still a lack of large prospective studies to evaluate its role in the treatment of HCC. Meanwhile, EASL Clinical Practice Guidelines [41] suggest that large prospective studies and especially randomized phase III trials are needed to support this therapy in the management of HCC.

Similar to other studies [11, 23, 42], our study showed that TACE-RFA is generally safe, and RFA did not increase the risk of death and the incidence of major complications. There was no significant difference in the incidence of major complications between TACE-RFA group and TACE group alone.

Retrospective and nonrandom design are the main limitations of our research. Therefore, a prospective randomized controlled trial is necessary to verify our results. Although there were statistical differences in the average tumor diameter and BCLC stage between the two groups, multivariate analysis showed that these two factors had no influence on OS and PFS of patients with BCLC stage A/B HCC.

## Conclusions

In summary, TACE combined with RFA treatment was maybe always beneficial to patients with BCLC A/B HCC regardless of tumor size, and these patients showed better benefits in terms of PFS and OS than those who had only received TACE. On the basis of our findings, the combination of TACE and RFA is likely to be a promising therapeutic option for these patients. However, further prospective randomized controlled trials are necessary to validate our observations.

## Data Availability

The data analysed during this study are avaliable from the electrical medical database of Union Hospital, Tongji Medical college, Huazhong University of Science and Technology. Please contact the author Chuansheng Zheng (hqzcsxh@sina.com) uponreasonable requests.

## Abbreviations

AFP: Alpha-fetoprotein
BCLC: Barcelona Clinic Liver Cancer
CIT: Carbon ion therapy
CPT: Charged particle therapy
CR: Complete response
CT: Computed tomography
EASL: European Association for the Study of the Liver
HCC: Hepatocellular carcinoma
MR: Magnetic resonance
mRECIST: Modified Response Evaluation Criteria in Solid Tumors
OS: Overall survival
PBT: Proton beam therapy
PD: Progressive disease
PFS: Progression-free survival
PR: Partial response
RFA: Radiofrequency ablation
SD: Stable disease
TACE: Transarterial chemoembolization
